# A role for ion homeostasis in yeast ionic liquid tolerance

**DOI:** 10.17912/micropub.biology.000718

**Published:** 2023-02-03

**Authors:** Lisa Liu, Rahim U. Ansari, Maikayeng Vang-Smith, Chris Todd Hittinger, Trey K. Sato

**Affiliations:** 1 DOE Great Lakes Bioenergy Research Center, University of Wisconsin-Madison, Madison, WI; 2 Department of Bacteriology, University of Wisconsin-Madison, Madison, WI; 3 Laboratory of Genetics, University of Wisconsin-Madison, Madison, WI; 4 Wisconsin Energy Institute, University of Wisconsin-Madison, Madison, WI; 5 J. F. Crow Institute for the Study of Evolution, University of Wisconsin-Madison, Madison, WI; 6 Center for Genomic Science Innovation, University of Wisconsin-Madison, Madison, WI

## Abstract

The model yeast *Saccharomyces cerevisiae* is being developed as a biocatalyst for the conversion of renewable lignocellulosic biomass into biofuels. The ionic liquid 1-ethyl-3-methylimidazolium chloride (EMIMCl) solubilizes lignocellulose for deconstruction into fermentable sugars, but it inhibits yeast fermentation. EMIMCl tolerance is mediated by the efflux pump Sge1p and uncharacterized protein Ilt1p. Through genetic investigation, we found that disruption of ion homeostasis through mutations in genes encoding the Trk1p potassium transporter and its protein kinase regulators, Sat4p and Hal5p, causes EMIMCl sensitivity. These results suggest that maintenance of ion homeostasis is important for tolerance to EMIMCl.

**Figure 1.**
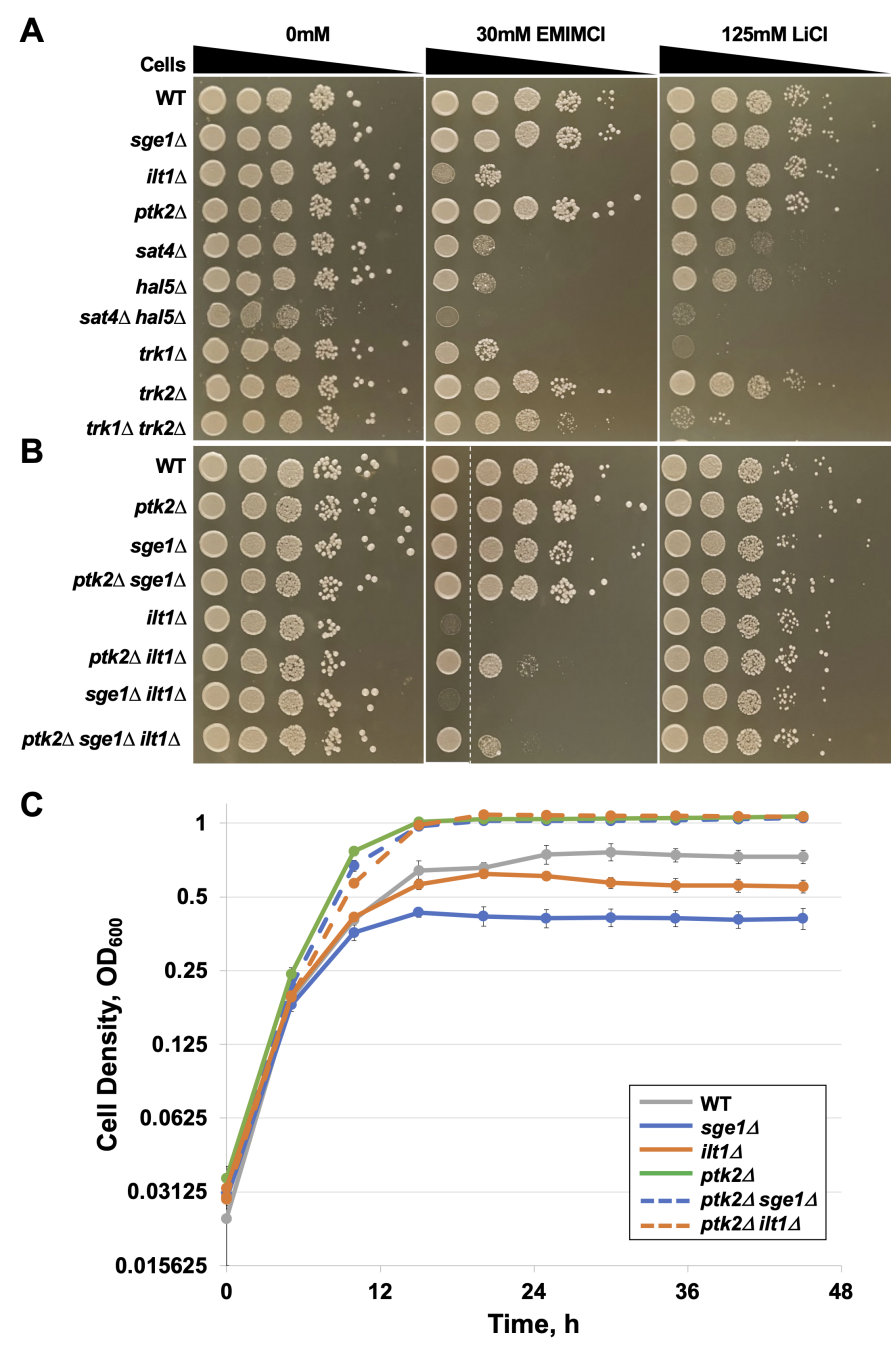
**Mutations in genes involved in ion homeostasis impact yeast EMIMCl tolerance. A-B)** Equivalent amounts of cells with the indicated genotypes were spotted on either YPD pH 5, YPD pH 5 with 30 mM EMIMCl, or YPD pH 5 with 125 mM LiCl. Each strain was serially diluted 1:10 six times, spotted, and imaged after 48 hours of growth at 30 ˚C. All spot assays were carried out in biological triplicate with two technical replicates each. All images were evaluated together, and a representative image of each plate was selected from all replicates. Any dashed white space indicates modification of image to align strains within the same plate image. **C)** Log phase cells were inoculated into YPD pH 5 with and without 125 mM EMIMCl at a cell density of 0.1 OD _600_ . Cell densities were measured every hour at OD _600_ for 48 hours at 30˚C. Growth experiments were carried out in biological triplicate. All datapoints from each replicate were averaged, and cell densities were graphed every 5 hours with standard error bars displayed over their respective datapoints.

## Description

In the past two decades, technological developments have encouraged our society to begin to shift from fossil fuels to biofuels made from renewable plant feedstocks (Liu et al. 2021). A major challenge with lignocellulosic plant materials is their recalcitrance to deconstruction into fermentable sugars. Biomass pretreatment with imidazolium ionic liquid (IIL) solvents, coupled with enzymatic hydrolysis, has been shown to be effective in producing glucose and xylose from switchgrass (Shi et al. 2013). IILs consist of a cationic imidazolium ring with different R groups paired with a monovalent anion, such as 1-ethyl-3-methylimidazolium chloride (EMIMCl). Although effective at biomass deconstruction, one challenge with using IILs, such as EMIMCl, is that they can remain at 150-270 mM in the hydrolyzed feedstocks after recovery (Ouellet et al. 2011; Li et al. 2013). These concentrations impair mitochondrial function and fermentation in the canonical biofuel-producing yeast *Saccharomyces cerevisiae* (Dickinson et al. 2016). This challenge calls for research into strategies to circumvent the toxicity that EMIMCl and other IILs have on *S. cerevisiae.*

Previously, we screened a yeast fosmid library for genes that are sufficient to increase the tolerance of *S. cerevisiae* to EMIMCl, and discovered that *SGE1* and *ILT1* are important for IIL tolerance (Higgins et al. 2018). Deletion of *SGE1* and *ILT1* each resulted in reduced yeast growth in the presence of 125 mM EMIMCl. *SGE1* encodes a plasma membrane-localized Major Facilitator Superfamily (MFS) efflux pump that removes toxic cations from the cell (Sá-Correia et al. 2009; dos Santos et al. 2014). The molecular function of the seven-pass transmembrane protein Ilt1 is unclear, but heterologous expression of *Yarrowia lipolytica* *ILT1* in *S. cerevisiae* conferred tolerance to IILs (Reed et al. 2019), which suggests that *ILT1* has a similar role in other yeast species.

As an alternative approach to identifying genes required for EMIMCl tolerance, we carried out two chemical genomic screens with a barcoded library of *S.* *cerevisiae* deletion mutants grown in the presence of individual inhibitory compounds that are commonly found in lignocellulose-derived hydrolysates, including medium containing EMIMCl (Dickinson et al. 2016; Vanacloig-Pedros et al. 2022). Our first study determined that deletion of *PTK2* , which encodes a serine-threonine protein kinase, resulted in enhanced tolerance to EMIMCl (Dickinson et al. 2016). In the second study, *sge1∆* and *ilt1∆* mutants from the library displayed reduced fitness in medium with EMIMCl relative to the total population of mutants, while *ptk2∆* mutants displayed greater fitness (Vanacloig-Pedros et al. 2022), which confirmed our previously published results (Dickinson et al. 2016). The second chemical genomic study also determined that deletion mutations in the paralogous *SAT4* and *HAL5* genes conferred significant fitness defects in EMIMCl medium, suggesting their potential roles in IIL tolerance (Vanacloig-Pedros et al. 2022). *SAT4* and *HAL5* encode serine-threonine kinases that regulate the plasma membrane localizations of MFS nutrient transporters (Tumolo et al. 2020) and the Trk1p/Trk2p potassium transporters (Mulet et al. 1999; Pérez-Valle et al. 2007), which are important for intracellular ion homeostasis. While phosphorylation of Sge1p has not been observed directly in biochemical assays, phosphoproteomic studies have identified phosphosites on the intracellular C-terminus of Ilt1p (Ficarro et al. 2002; Holt et al. 2009; Soulard et al. 2010; Swaney et al. 2013; MacGilvray et al. 2020; Lanz et al. 2021). Together, these results led us to hypothesize that the Sat4p, Hal5p, and Ptk2p protein kinases may function in IIL tolerance by regulating the phosphorylation of Ilt1p and Sge1p.

To confirm the requirements for *SAT4* and *HAL5* in EMIMCl tolerance, we constructed and compared the growth of *S. cerevisiae* strains harboring deletion mutations in *SGE1* , *ILT1* , *SAT4* , *HAL5* , and *PTK2* in spot assays. On 30 mM EMIMCl, both *sat4∆* and *hal5∆* mutants displayed reduced cell growth to a degree similar to the *ilt1∆* mutant strain, ( **Fig. 1A** ), while they grew similarly to wild-type (WT) cells on medium lacking EMIMCl. The *sat4∆ hal5∆* double mutant displayed extreme sensitivity to EMIMCl, but this strain also grew slowly on the control medium. *SAT4* and *HAL5* were previously shown to regulate *TRK1* function and necessary for tolerance to the Li ^+^ cation (Mulet et al. 1999; Pérez-Valle et al. 2007), which impairs cell growth, likely by competing with the uptake of essential K ^+^ cations (Bertl et al. 2003). Therefore, we generated *trk1∆* and *trk2∆* mutants and tested all strains for growth on 125 mM LiCl in the spot assays. Consistent with published results (Mulet et al. 1999; Erez and Kahana 2002; Bertl et al. 2003; Pérez-Valle et al. 2007), the *sat4∆,* *hal5∆* , and *trk1∆* single and *trk1∆ trk2∆* double mutants grew slower than the WT strain on LiCl ( **Fig. 1A** ). Deletion of *TRK2* had a modest effect on growth on LiCl, which was also observed elsewhere (Bertl et al. 2003), and an unexpected beneficial effect on EMIMCl when combined with the *trk1∆* mutation. Interestingly, the *trk1∆* mutant strain grew poorly on EMIMCl (similar to the *ilt1∆* mutant), while the *ilt1∆* mutant grew similar to the WT strain on LiCl. This result indicated that Ilt1p does not function in general monovalent cation influx or ion homeostasis and instead plays a more specific role in tolerance to IILs and related compounds. While these results suggest that Trk1p could also function as an EMIM ^+^ efflux pump, a more plausible interpretation is that Trk1p, along with Sat4p and Hal5p, plays an indirect but important role in EMIMCl tolerance through ion homeostasis. Additional experimentation will be needed to conclusively dissect these mechanisms.

Our results suggest that cellular functions other than direct extrusion of toxins play important roles in EMIMCl tolerance. In addition to its role in IIL tolerance, Ptk2p is serine-threonine kinase that regulates ion homeostasis through the plasma membrane ATPase Pma1p (Eraso et al. 2006), and deletion of *PTK2* confers tolerance to toxic polyamines (Erez and Kahana 2002). In contrast to the phenotypes of the *sat4∆* and *hal5∆* mutants, *ptk2∆* causes a fitness benefit over the wild-type strain in the presence of EMIMCl ( **Fig. 1A-B** ). We further found that *ptk2∆* had little effect on LiCl tolerance ( **Fig. 1A-B** ), a phenotype shared by the *sge1∆* and *ilt1∆* mutants. This suggests the possibility that Ptk2p may negatively regulate Sge1p and Ilt1p activities via phosphorylation. We tested this possibility by conducting epistasis experiments using strains harboring combinations of deletion mutations in *PTK2* , *SGE1* , and *ILT1* . The *ilt1∆* single and *sge1∆ ilt1∆* double mutants did not grow in the presence of 30 mM EMIMCl, but the *ptk2∆ ilt1∆* double and *ptk2∆ sge1∆ ilt1∆* triple mutants displayed noticeable growth ( **Fig. 1B** ). Deletion of *SGE1* had little effect on growth in the presence of 30 mM EMIMCl, which obscured our ability to test for epistasis with *PTK2* . Therefore, we tested their genetic interactions by culturing in liquid medium containing 125 mM EMIMCl, the identical condition in which we previously detected a requirement for *SGE1* and *ILT1* in IIL tolerance (Higgins et al. 2018). In 125 mM EMIMCl, the *sge1∆* and *ilt1∆* single deletions displayed slower growth and reached a lower final cell density than the WT strain ( **Fig. 1C** ). In contrast, *ilt1∆ ptk2∆* and *sge1∆ ptk2∆* double mutants phenocopied the *ptk2∆* single mutant, wherein all three strains displayed significantly faster growth and reached higher final cell densities than the WT, *sge1∆* , and *ilt1∆* single mutant strains. These results indicate that Ptk2p does not function exclusively with Sge1p and Ilt1p in EMIM ^+^ tolerance; instead, Ptk2p may regulate other undetermined efflux pumps that can export EMIM ^+^ from the cytosol. Interestingly, others have reported that *ptk2∆* *trk1∆ trk2∆* triple mutant cells had increased polyamine tolerance and hypersensitivity to LiCl (Erez and Kahana 2002). Taking all of the results into account, we conclude that role of Ptk2p in EMIMCl tolerance is most likely through the regulation of general ion homeostasis, rather than solely through the direct regulation of Sge1p and Ilt1p efflux activities.

A better understanding of the genetic basis of EMIMCl tolerance may expedite the development of a sustainable lignocellulosic biofuel industry that utilizes IIL pretreatment in its processing pipeline. Our results indicate that tolerance to EMIMCl is complex and involves the coordination of efflux pumps and ion homeostasis, as well as providing new potential target genes for engineering IIL-tolerant *S. cerevisiae* strains for the biofuel industry.

## Methods

*Media and Culturing Conditions*

*S. cerevisiae* strains were cultured as described previously (Sherman 2002). Sterilized standard YPD medium was used for overnight culturing and YPD at pH 5 for subculturing, specifically 10 g/L yeast extract, 20 g/L peptone, and 20 g/L dextrose (D-glucose) with appropriate antibiotic; if needed, pH was adjusted with HCl. For solid agar plates, 25 g/L of agar were added to media prior to autoclave sterilization. As necessary, media were supplemented with 200 µg/ml Geneticin (G418; cat#G1000, US Biological), 200 µg/ml hygromycin B (Hyg; cat#H9700-05B, US Biological), and/or 200 µg/ml Zeocin (Zeo; cat#R25001, Invitrogen).

For spot assays, 2x YPD at pH 5 medium supplemented, as needed, with 60 mM EMIMCl (cat#272841, Sigma-Aldrich) or 250mM LiCl (cat#7447-41-8, Calbiochem) was filter sterilized into a 50 g/L autoclaved agar mixture so that all components of the medium were at 1x working concentration. The final sterile 1x agar medium was well mixed before aliquoting into petri dishes and cooled for 48 hours.

*Yeast Strain Engineering*

Strains used in this study are described in **Table 1** . Yeast transformations were conducted by following the heat shock and lithium acetate method (Schiestl and Gietz 1989). For integration of linear DNA, primers containing 40-60 bp of homologous sequences that flank targeted genes in the *S* . *cerevisiae* genome were amplified by Polymerase Chain Reaction (PCR) with *LoxP-KanMX-LoxP* (from pUG6; (Guldener et al. 1996)) or *LoxP-HphMX-LoxP* (from pUG75; (Hegemann and Heick 2011)) as DNA templates. To excise the antibiotic marker via flanking LoxP sites, 0.1-1 µg pSH65 plasmid, which encodes a galactose-inducible Cre recombinase, was transformed into the strain and used according to published protocols (Gueldener et al. 2002)

To confirm all strain engineering, transformed candidates were grown independently in YPD liquid medium, and genomic DNA was extracted (cat#MPY80200, Lucigen). For sequence verification, sites of genetic modification were amplified by PCR with primers that flank the insertion site, purified, and then Sanger-sequenced by University of Wisconsin-Madison Biotechnology Center.

*Spot Assays*

Saturated cultures were subcultured to allow for 1-2 generations of growth in fresh YPD adjusted to pH5. Culture densities were measured; then, cells were harvested, washed, and normalized to 1 optical density at 600 nm (OD _600_ ) with sterile double-distilled water. Cell suspensions were serially diluted 1:10 in a 96-well plate, and 4 µl of the suspension was pipetted onto YPD at pH5 +/- 30 mM EMIMCl or 125 mM LiCl solid media. EMIMCl or LiCl concentrations were optimized to facilitate the observation of dynamic fitness differences. Experiments were performed in independent biological triplicate with two technical replicates for each strain and condition. YPD agar plates adjusted to pH 5 were included as loading control. All spot assay plates were incubated for 48 hours at 30 ºC and then imaged. All images were envaulted together, and a representative image of each plate was selected from all replicates. Any dashed white line indicates modification of the image to align strains within the same plate image.

*24-Well Growth Assays*

Overnight cultures were subcultured in YPD at pH5 for 1-2 doublings. Log phase cells were harvested, washed, and resuspended with sterile double-distilled water. Cells were inoculated to 0.1 OD _600_ /ml in 24 well microtiter plate with 1.5 ml YPD at pH 5 +/- 125 mM EMIMCl. Cell concentrations were measured every hour by OD _600_ for 48 hours by a Tecan Infinite M200 Pro with continuous shaking. Each experiment was conducted in biological triplicate, and standard error bars are displayed over each datapoint. All datapoints from each replicate were averaged; then, cell densities were graphed every 5 hours with standard error bars displayed over their respective curve.

## Reagents

Table 1. *S. cerevisiae* strains used in this study.

**Table:** 

**Strain Name**	**Genotype**	
BY4741	*MAT* **a** *his3Δ1 leu2Δ0 met15Δ0 ura3Δ0*	(Brachmann et al. 1998)
GLBRCY386	BY4741 *ilt1∆::KanMX*	(Higgins et al. 2018)
GLBRCY430	BY4741 *sge1∆::LoxP*	(Higgins et al. 2018)
GLBRCY547	BY4741 *ptk2∆::LoxP-HygMX*	This study
GLBRCY549	BY4741 *sge1∆::LoxP ptk2∆::LoxP-HygMX*	This study
GLBRCY568	BY4741 *sge1∆::LoxP ilt1∆::LoxP-KanMX*	This study
GLBRCY722	BY4741 *sge1∆::LoxP ilt1∆::LoxP ptk2∆::LoxP-HygMX*	This study
GLBRCY734	BY4741 *ptk2∆::LoxP-KanMX ilt1∆::LoxP-HygMX*	This study
GLBRCY1037	BY4741 *sat4∆::LoxP-KanMX*	This study
GLBRCY1066	BY4741 *hal5∆::KanMX*	This study
GLBRCY1086	BY4741 *sat4∆::LoxP-KanMX hal5∆::LoxP-HygMX*	This study
GLBRCY1087	BY4741 *trk2∆::KanMX*	This study
GLBRCY1130	BY4741 *trk1∆::LoxP-HygMX trk2∆::LoxP-KanMX*	This study
GLBRCY1171	BY4741 *trk1∆::LoxP-HygMX*	This study
